# 2021 Electrophysiology Literature in Review: A Surgeon’s Perspective

**DOI:** 10.19102/icrm.2022.130106

**Published:** 2022-01-15

**Authors:** Andy C. Kiser

**Affiliations:** ^1^Department of Cardiothoracic Surgery, University of Pittsburgh School of Medicine, Pittsburgh, PA, USA; ^2^St. Clair Health, Pittsburgh, PA, USA

**Keywords:** Atrial fibrillation, bi-atrial maze, hybrid ablation, left atrial appendage occlusion

The 2021 literature reflects important milestones for surgical arrhythmia management. The published outcomes from the Convergence of Epicardial and Endocardial Ablation for the Treatment of Symptomatic Persistent Atrial Fibrillation (CONVERGE) trial supported the hybrid convergent procedure over catheter ablation alone for patients with persistent and long-standing persistent atrial fibrillation (AF).^1^ The trial further reinforced the advantages of collaboration between surgeons and electrophysiologists in the management of AF. The Left Atrial Appendage Occlusion Study III (LAAOS III) trial provided critical evidence that left atrial (LA) appendage (LAA) occlusion during open cardiac surgical procedures reduces the risk of stroke in patients entering the operating room with AF.^2^ A provocative retrospective cohort analysis found an increased risk of bleeding complications after coronary artery bypass surgery (CABG) for patients discharged on anticoagulation (AC) for new-onset AF.^3^ The report challenges guideline-directed therapy for this select cohort of post-cardiotomy AF patients. Finally, a very interesting Bayesian network meta-analysis examined lesion sets for the surgical treatment of AF.^4^ The report found no difference in AF recurrence for patients undergoing a bi-atrial lesion pattern over those with lesions limited to the LA. This summary reviews these important publications from 2021.

## Convergent hybrid ablation benefits patients with persistent and long-standing persistent atrial fibrillation

The CONVERGE trial^1^ prospectively randomized 153 patients from 27 centers, including 25 in the United States (US) and 2 in the United Kingdom, with persistent and long-standing persistent AF who were symptomatic and refractory to drug therapy. Patients were randomized 2:1 to the hybrid convergent arm or to the endocardial catheter ablation arm, respectively. The hybrid convergent lesion pattern included surgical, unipolar radiofrequency (RF) ablations performed via a transdiaphragmatic or subxiphoid approach. Surgeons created ablation lines at the right and left epicardial antrum and contiguously parallel ablation rows along the posterior LA. Endocardial mapping followed the surgical portion to complete the pulmonary vein isolation (PVI) with an irrigated RF catheter to close any breakthrough gaps in the surgical lesions and created a cavotricuspid isthmus line. The electrophysiologist confirmed PVI by demonstrating entrance and exit block. The catheter ablation group underwent right and left PVI with a connecting roof line using irrigated RF. The electrophysiologist also confirmed PVI by documenting the entrance and exit block and created a cavotricuspid isthmus line with bi-directional block. Follow-up included 24-hour Holter monitoring at 6 and 12 months and seven-day Holter monitoring at 18 months.

After a 90-day blanking period, the hybrid convergent cohort demonstrated 53.5% freedom from atrial arrhythmias from 3 to 12 months without anti-arrhythmic drugs (AADs) compared to 32.0% freedom from atrial arrhythmias in the catheter ablation cohort (P = .0128). When cohorts were analyzed with or without AADs, 76.8% of the hybrid convergent group were free from atrial arrhythmias compared to 60.0% of the catheter ablation group (P = .0329). Outcomes from the seven-day Holter monitoring at 18 months demonstrated at least a 90% reduction in AF burden for the hybrid convergent cohort, while the catheter ablation cohort showed a 55% reduction in AF burden (P = .0395).

None of the patients experienced death, esophageal fistula, or cardiac perforation. Eight major adverse events were reported for the hybrid convergent group, with none reported for the catheter ablation group. Three events (1 stroke, 1 excessive bleeding, and 1 excessive bleeding with pericardial effusion) occurred within 7 days of the procedure, while 5 events (3 pericardial effusions, 1 phrenic nerve injury, and 1 transient ischemic attack) occurred between 7 and 30 days post-procedure. The 3 late pericardial effusions were delayed inflammatory responses to the intra-pericardial surgical procedure, not cardiac perforations, and were managed with elective pericardial fluid drainage.

The CONVERGE trial represents the first multicenter, prospective, randomized trial comparing the effectiveness of a hybrid endocardial and epicardial ablation procedure to endocardial catheter ablation for patients with persistent AF regardless of the duration of AF. As a result of this trial, the convergent procedure has received US Food and Drug Administration approval for the treatment of patients with long-standing persistent AF. The CONVERGE trial further emphasizes the value of a collaborative, multidisciplinary treatment for patients with non-paroxysmal AF.

## Concomitant left atrial appendage occlusion does reduce stroke rate

The LAAOS III investigators^2^ performed a prospective, multicenter, randomized trial comparing patients with AF undergoing cardiac surgery for other indications who further underwent LAA occlusion (2,379 patients) to those who did not receive LAA occlusion (2,391 patients). All patients had a CHA_2_DS_2_-VASc score of >1 point (mean, 4.2 points) and were followed up for an average of 3.8 years post-procedure. The LAA occlusion technique included all surgical techniques and commercially available means of occlusion or exclusion, except percutaneous or purse-string closure. Of note, the data and safety monitoring board recommended stopping the trial early due to efficacy outcomes.

There were no significant differences in procedural or hospitalization outcomes between the two groups. AC at discharge was prescribed in 83.4% of the occlusion group and in 81.0% of the control group with corresponding AC use in 79.6% and 78.9% at 1 year and 75.3% and 78.2% at 3 years. Ischemic stroke or systemic arterial embolization occurred in 4.8% of patients in the occlusion group compared to 7.0% in the non-occlusion group (P = .001). Of interest, the occurrence of an ischemic stroke or systemic arterial embolization did not differ between the two cohorts within 30 days of the procedure (2.2% for occlusion, 2.7% for non-occlusion). The significant difference between groups occurred after 30 days (2.7% for occlusion, 4.6% for non-occlusion), being attributed the improved stroke and arterial embolization rates to a reduction in cardiac thromboembolization originating from the LAA.

The authors summarized their results by concluding that LAA occlusion provides an additive improvement over AC alone for the prevention of stroke and arterial embolization. The study did not compare LAA occlusion without AC to AC alone without LAA occlusion. The trial, therefore, does not provide evidence that LAA occlusion replaces the need for AC. However, for patients with AF undergoing cardiac surgery, the study provides ample evidence that surgeons should address the LAA concomitantly.

## Bleeding risk supports surgeons’ preference to avoid anticoagulation and use only amiodarone for postoperative atrial fibrillation

A retrospective analysis of the Society of Thoracic Surgeons Adult Cardiac Surgery Database was performed to evaluate the management of patients undergoing isolated, first-time CABG who developed postoperative AF.^3^ This study examined the practice patterns of cardiac surgeons in the US and Canada regarding their treatment strategy and divided them into the following cohorts: no AC/no amiodarone (AR), no AC/AR, AC/no AR, or AC/AR. The outcomes focused on 30-day readmission for stroke or readmission for any bleeding event. Overall, 166,747 patients met the analysis inclusion criteria, with 29,150 in the no AC/no AR group, 94,755 in the no AC/AR group, 8,672 in the AC/no AR group, and 34,170 in the AC/AR group. Despite minor regional treatment variability, the groups had similar baseline clinical characteristics.

Treatment patterns tended to align with preoperative CHA_2_DS_2_-VASc score. Patients with a higher CHA_2_DS_2_-VASc score were more likely to receive AC and less likely to receive AR (when CHA_2_DS_2_-VASc score was 0 points, 16.4% received AC and 79.3% received AR; when CHA_2_DS_2_-VASc score was 5–9 points, 29.9% received AC and 73.5% received AR). While the overall readmission rate for stroke or bleeding was <1%, the readmission rate for bleeding was significantly higher for patients discharged on AC (their adjusted odds ratio [aOR] was 4.3; P = .0001), with no difference in stroke readmission regardless of treatment with or without AC (P = .571). There was, however, an association between AC/AR therapy and lower stroke readmission (aOR, 0.65; P = .015) compared to AR alone (in 56.8% of patients). The impact of AR on international normalized ratio for warfarin-treated patients did not contribute to an increased bleeding risk.

Despite our medical and surgical societies’ guidelines recommending AC for postsurgical patients with AF, the majority of patients discharged after CABG with AF received AR therapy alone and did not receive AC. Even when the CHA_2_DS_2_-VASc score was ≥5 points, <30% of patients received AC in this analysis. The four-fold increase in bleeding with no difference in the aOR for 30-day stroke readmission challenges the guidelines and seems to support the treatment philosophy employed by most cardiac surgeons in the US and Canada.

## Do all atrial fibrillation patients need a full bi-atrial maze procedure?

A review article this year performed a Bayesian network meta-analysis of published randomized controlled trials (RCTs) for patients undergoing concomitant surgical PVI, left atrial maze (LAM), bi-atrial maze (BAM), or no concomitant AF ablation. The study explored one-year differences in freedom from AF, early mortality, and permanent pacemaker implantation (PPMI). Nineteen RCTs produced 2,031 patients who qualified for inclusion in this analysis (248 patients with PVI, 599 with LAM, 458 with BAM, and 726 with no ablation). Only 10% (203) of patients had paroxysmal AF, while the remaining 90% (1,828) had persistent or long-standing persistent AF. Follow-up was completed at a minimum of 1 year with 12-lead electrocardiography, 24-hour Holter monitoring, or an implanted monitoring device.

This network meta-analysis set out to effectively reduce the inherent behavior bias of surgeons found in a pairwise meta-analysis. The analysis revealed overall freedom from AF rates of 67.25% for PVI, 69.43% for LAM, 75.12% for BAM, and 28.08% for no ablation. Although the fixed-effects model demonstrated a significantly higher rate of freedom from AF for the BAM over PVI in the Bayesian comparison, the random-effects model did not show a significant benefit between the three ablation approaches. BAM did demonstrate a higher mortality rate than no ablation, while the rate of mortality or PPMI did not differ between the ablation groups.

The limitations of this study included small sample sizes, variable procedural techniques, and questionable accuracy of follow-up monitoring, which impacted the reliability of this analysis. The authors recognized these and other limitations, suggested that right atrial pathology may warrant BAM, and recognized that PVI remains the cornerstone of ablation therapy for all lesion patterns. However, in non-selected patients with AF, BAM did not demonstrate improved outcomes over PVI or LAM and, in addition, BAM may increase mortality over no ablation.

## Author’s comments

The CONVERGE trial provides a long-awaited comparison of the hybrid convergent procedure to catheter ablation alone. The results, particularly for those patients with long-standing persistent AF, encourage a more collaborative management strategy for this patient population. While pericardial effusion was the most common adverse event, complications are generally related to the inflammatory process of intra-pericardial surgery and may be managed electively. We should, however, continue to follow events such as cerebral vascular accidents and phrenic nerve palsy as well as esophageal injury and death as we evaluate ongoing clinical data.

AR therapy, even prophylactically, has gained much enthusiasm among cardiac surgeons as we strive to reduce the burden and consequences of postoperative AF. We find from this large retrospective analysis that most surgeons treat postoperative AF with AR and, often, avoid AC despite guideline recommendations. The incidence of bleeding among patients discharged on AC noted above lends credibility to the surgeon’s decision. One should contemplate, however, the patient’s CHA_2_DS_2_-VASc score as a guide when weighing the bleeding and stroke risks as there was a trend toward stroke risk reduction with AC in the higher-risk patient population.

A survey of surgeons in 2010 by the American Association of Thoracic Surgeons identified reasons why surgeons do not address AF during concomitant cardiac surgical procedures. The top five reasons were: AF surgery is too complex; AF surgery increases pump time; academic results are not reproducible in private practice; AF surgery increase operative risk; and there is a lack of consensus opinion on patient selection, lesion pattern, and energy sources. We must address the surgeons’ apprehension through education and mentorship. LAA management is perhaps the most opportune first step in developing a surgical AF management strategy. LAA occlusion or removal during concomitant cardiac surgery not only reduces the stroke risk, as evidenced by the LAAOS III investigators, but also serves as an introduction to surgical AF therapies while creating enthusiasm for more definitive AF procedures. Not all patients need a full BAM procedure to benefit from a concomitant AF ablation. The Bayesian network meta-analysis lends evidence to the benefit of PVI and LAM for patients with non-paroxysmal AF. Progressive, step-wise learning, which starts with LAA management, builds experience and enables the surgeon to develop increasing procedural confidence, which eventually offers treatment alternatives to more patients with concomitant AF.
